# Health transformation toward universal healthcare coverage amidst conflict: examining the impact of international cooperation in Niger

**DOI:** 10.3389/fpubh.2024.1303168

**Published:** 2024-03-07

**Authors:** Mahamadou Doutchi, Abdoulaye Ghousmane, Fatimata Zampaligre, Bizo Moussa, El Khalef Ishagh, Ouédraogo Talatou Marc, Batouré Oumarou, Mutenda Sheria Kaya, Gbaguidi Aichatou Diawara, Abdoulaye Macire Camara, Seyni Moussa, Kuyangisa Bienvenu, Joseph Toko, Hamidou Harouna, Haladou Moussa, N’Zue Kofi, Jacques Lukenze Tamuzi, Patrick D. M. C. Katoto, Charles S. Wiysonge, Blanche-Philomene Melanga Anya

**Affiliations:** ^1^Department of Public Health, Université de Zinder, Zinder, Niger; ^2^Bureau de l'Organisation Mondiale de la Santé (OMS), Niamey, Niger; ^3^Direction de la Surveillance et la Réponse aux Epidémies du Ministère de la Santé Publique, de la Population et des Affaires Sociale, Chargé de la Surveillance, Niamey, Niger; ^4^Division of Epidemiology and Biostatistics, Department of Global Health, Faculty of Medicine and Health Sciences, Stellenbosch University, Cape Town, South Africa; ^5^Office of the President and CEO, South African Medical Research Council, Cape Town, South Africa; ^6^Centre for Tropical Diseases and Global Health, Department of Medicine, Catholic University of Bukavu, Bukavu, Democratic Republic of Congo; ^7^Cochrane South Africa, South African Medical Research Council, Cape Town, South Africa; ^8^World Health Organization Regional Office for Africa, Brazzaville, Republic of Congo

**Keywords:** international cooperation, armed conflicts, health system, transformation, partners, Niger

## Abstract

**Background:**

Approximately 70% of Sub-Saharan African countries have experienced armed conflicts with significant battle-related fatalities in the past two decades. Niger has witnessed a substantial rise in conflict-affected populations in recent years. In response, international cooperation has aimed to support health transformation in Niger’s conflict zones and other conflict-affected areas in Sub-Saharan Africa. This study seeks to review the available evidence on health interventions facilitated by international cooperation in conflict zones, with a focus on Niger.

**Methods:**

We conducted a systematic literature review (SLR) adhering to the Preferred Reporting Items for Systematic Reviews and Meta-Analyses (PRISMA) guidelines. The search was conducted from 2000 to 4 September 2022 using MeSH terms and keywords to identify relevant studies and reports in Sub-Saharan Africa and specifically in Niger. Databases such as PubMed (Medline), Google Scholar, Google, and gray literature were utilized. The findings were presented both narratively and through tables and a conceptual framework.

**Results:**

Overall, 24 records (10 studies and 14 reports) that highlighted the significant role of international cooperation in promoting health transformation in conflict zones across Sub-Saharan Africa, including Niger, were identified. Major multilateral donors identified were the World Health Organization (WHO), United Nations Children’s Fund (UNICEF), United Nations Fund for Population Activities (UNFPA), World Bank, United States Agency for International Development (USAID), European Union, European Commission Humanitarian Aid (ECHO), Global Fund, and Global Alliance for Vaccines and Immunization (GAVI). Most supports targeted maternal, newborn, child, adolescent, and youth health, nutrition, and psycho-social services. Furthermore, interventions were in the form of public health initiatives, mobile clinic implementation, data management, human resource capacity building, health information systems, health logistics, and research funding in conflict zones.

**Conclusion:**

This literature review underscores the significant engagement of international cooperation in strengthening and transforming health services in conflict-affected areas across Sub-Saharan Africa, with a particular focus on Niger. However, to optimize the effectiveness of healthcare activities from short- and long-term perspectives, international partners and the Ministry of Public Health need to re-evaluate and reshape their approach to health intervention in conflict zones.

## 1 Background

In recent decades, many countries in sub-Saharan Africa (SSA) have experienced civil or intrastate wars, as well as, to a lesser extent, foreign warfare. Armed conflicts have happened more frequently in SSA than in other parts of the world. Out of SSA, Ukraine, Palestine, and many other countries are facing humanitarian crises related to armed conflicts severely impacting health systems. An estimated 70% of all countries in SSA have witnessed an armed conflict since 1980 (1) and at least 1,000 battle-related direct deaths per year in the past two decades (2–4). These armed conflicts negatively impact the supply of care by limiting access to healthcare in front-line health centers, as most of the health systems in SSA are fragile (5). The increase in the number of armed attacks, mainly associated with the activities of armed terrorist groups, has reduced the population’s access to basic health services in SSA (6, 7). The high prevalence of armed conflict in SSA and its adverse effects on the delivery of maternal health services by health systems could be one of the reasons why the region also has the highest number of maternal childbirth deaths in the world (8). The healthcare system continues to be heavily affected by the security situation in SSA, particularly in Sahel countries. Niger reached record maternal and child mortality rates in 2018 and 2019 (9). In 2021, Niger experienced four epidemic health crises: measles which has been raging for 3 consecutive years; the coronavirus disease 2019 (COVID-19), present since 2020, and two other epidemics of meningitis and cholera, which affected the country after 2 years of calm. In 2022, Niger also experienced its first diphtheria epidemic in the department of Gouré, a pivotal area hosting people displaced by the Boko Haram conflict (10). In addition, this violence has impacted the living conditions of Nigeriens, reduced their living space, and led to internal and external migrations (11). Added to this are the hazards linked to climate change, such as floods, infectious diseases, and drought in Niger. The instability of the security situation constitutes a threat to territorial integrity, national cohesion, and the promotion of a healthy and productive population. Access to basic social services, including health, education, and water, is a real challenge for these populations (12). Since 2015, kidnappings of health workers and acts of violence against them in primary healthcare centers and dispensaries have been regularly reported (13–15). Violence due to armed conflict has created physical and mental disabilities and disrupted health systems. There are also significant medico-legal, bioethical, and social difficulties surrounding armed conflicts. Access to abortion for women who have been raped is a significant issue that has arisen as a result of armed conflicts (16). Access to healthcare deteriorated as essential drug supply chains were interrupted and health workers abandoned their posts (13). The number of people affected by these armed conflicts has reached unprecedented levels in recent years in the country (2, 11). According to the overview of humanitarian needs in Niger for the year 2022, around 2.4 million people will be food insecure, and around 1.23 million will have health needs (17). By the way, the budget required to achieve this target is estimated at USD 552.6 million through 148 operational partners (9). However, the budget allocated to the Ministry of Health remains lower, while the contribution of households to health expenditure remains higher in the country. The country’s humanitarian action plan for 2021 only provides funding equivalent to 42% of the needs expressed by stakeholders.

To address these shortcomings, Niger, through bilateral or multilateral cooperation, benefits from *ad hoc* support in strengthening the local health system. The government of Niger, with the support of international partners, is on the front lines to meet these health challenges. Various forms of technical and financial support for curative and preventive healthcare have supported the health system in the conflict zones of Niger (9, 13, 18). This support often takes the form of financial assistance through the Ministry of Health or international organizations. However, the impact of this international cooperation in support of health transformation in conflict zones is less studied in SSA in general and in Niger in particular. This study aims to review the available evidence on health transformation interventions through international cooperation in the conflict zones in SSA in general and in Niger in particular.

## 2 Methods

This was a desk review including studies and reports on health interventions implemented in conflict zones by non-governmental organizations (NGOs) or organizations with international funding in Sub-Saharan Africa and Niger, respectively. A systematic literature review (SLR) was carried out to answer this question, and the planned method for reporting systematic reviews and meta-analyses (PRISMA) was used (19). This method improved the transparency and consistency of the reporting of this SLR literature.

### 2.1 Eligibility criteria

The first phase includes all reports from foreign non-governmental organizations, with the following highlighted: (i) confit zones in Niger; (ii) non-governmental organizations (NGOs) and organizations active in health transformation in Niger, particularly in conflict zones; and (iii) documents and reports containing health-related results. Several online sources were used to identify conflict zones in Niger. The DEP (direction of research and programming) of the Ministry of Health, Population, and Social Affairs was consulted to identify NGOs working in the health field. Finally, to identify the reports, official sources such as the focal points of the ministries of health and other relevant administrative bodies, the United Nations, and NGOs were consulted. These documents included national health strategies, tools designed for health and nutrition crisis response interventions, national nutrition and health surveys, operational plans, and health system diagnostic reports. The targeted stakeholders include all actors involved in assisting the health sector; all technical and financial partners involved have already been identified by the Ministry of Public Health, Population and Social Affairs (MSP/P/AS). The second stage is the analysis of these reports through critical reading and comparison with data from the literature. A systematic review search method was conducted to find potential studies through the database. All types of study designs conducted in SSA describing international cooperation interventions in supporting health transformation in conflict zones were included. We included studies conducted from 01 January 2001 to 4 September 2022 without restriction on the language. Reports that included international cooperation but did not highlight health transformation in Niger were excluded from the review. Similarly, research conducted in SSA in which international cooperation did not include health transformation in SSA conflict zones was also ruled out. Finally, articles presented at conferences and congresses, as well as those that did not indicate beneficial interventions, were excluded.

### 2.2 Search strategy

The databases were consulted from 01 January 2001 to 01 September 2022 using a literature review approach, including (i) reference documents and reports: National Health Policy/Health Sector Strategy version 2015; Health Development Plan No. 3 (PDS 2017–2021); United Nations Development Assistance Framework Plan (UNDAF 2019–2021); national health accounts; demographic health surveys (EDS-MICS); national health financing strategy 2012; (ii) Several data sources were used, including PubMed (Medline), Google Scholar, World Health Organization (WHO), the Niger Ministry of Health, the European Union, and annual reports from non-governmental organizations. The search strategy was based on key words and MeSH terms related to health interventions in the context of international cooperation in conflict areas. The following search strategy was used: “International cooperation” OR “Foreign Aid” OR “Treaties” OR “Treaty” OR “Medical Missions” AND “Armed Conflict” OR “Armed Conflicts” OR “War” OR “Wars” AND “health systems plans” OR “health systems plan” OR “Health Planning” OR “Comprehensive Health Plans” AND “universal health coverage” OR “universal coverage” OR UHC” OR AND “Cameroon” OR “Central African Republic” OR “Chad” OR “Congo” OR “Democratic Republic of the Congo” OR “Equatorial Guinea” OR “Gabon” OR “Sao Tome and Principe” OR “Burundi” OR “Comoros” OR “Djibouti” OR “Eritrea” OR “Ethiopia” OR “Kenya” OR “Madagascar” OR “Rwanda” OR “Seychelles” OR “Somalia” OR “South Sudan” OR “Sudan” OR “Tanzania” OR “Uganda” OR “Angola” OR “Botswana” OR “Eswatini” OR “Lesotho” OR “Malawi” OR “Mozambique” OR “Namibia” OR “South Africa” OR “Zambia” OR “Zimbabwe” OR “Benin” OR “Burkina Faso” OR “Cabo Verde” OR “Cote d’Ivoire” OR “Gambia” OR “Ghana” OR “Guinea” OR “Guinea-Bissau” OR “Liberia” OR “Mali” OR “Mauritania” OR “Niger” OR “Nigeria” OR “Senegal” OR “Sierra Leone” OR “Togo.”

A librarian was consulted to verify and approve the search strategy used. The other documents were compiled with officials from the MSP/P/AS, NGO leaders, international organizations, and other sources.

## 3 Results

**Figure 1:**
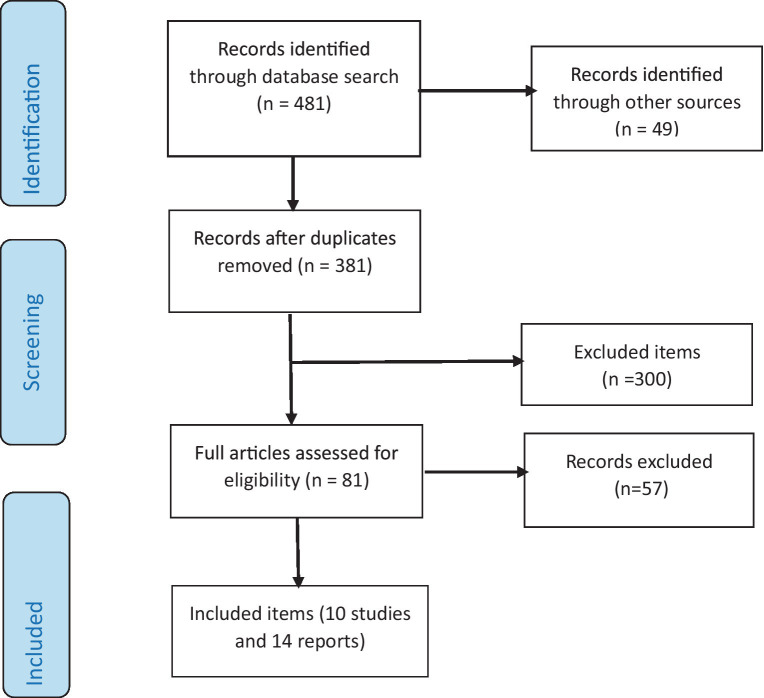
Flowchart depicting selected studies and reports. Adapted with permission from (19), licensed under CC BY 4.0.

A total of 481 articles were identified through PubMed (Medline), Google Scholar, Google, and the sites of United Nations system agencies and international organizations. After removing duplicates, 381 articles were screened by title and abstract. Among them, 300 articles were eliminated because they did not meet the inclusion criteria. After a careful review of the full-text articles, 74 were excluded because they did not meet the inclusion criteria described above. The systematic review ultimately focused on 10 articles and 14 reports, which were used for a narrative synthesis. Figure 1 shows the selection process for articles and reports identified in this review.

### 3.1 Characteristics of articles and activity reports

Table 1 describes the five studies included in terms of first author and country, population included in the study, highlights, international interventions, and study objectives. Among the 10 studies included, seven assessed international interventions in support of healthcare during the conflict, and the other three studies were conducted after the conflict. There were 10 studies that described international cooperation interventions in enabling health transformation in sub-Saharan conflict zones. Ethiopia, Niger, Mali, Burkina Faso, Democratic Republic of the Congo, Central African Republic, Nigeria, South Sudan, Kenya, Somalia, Mozambique, Eritrea, Rwanda, Burundi, Mali, Kenya, Uganda, and Zimbabwe were the primary study countries. Table 2 describes 14 reports consulted, among which are the following: Ministry of Public Health (1 report), ICRC (International Committee of the Red Cross) (1 report), Médecins Sans Frontières (MSF) (3 reports), Save the Children (2 reports), Action Against Hunger (ACF) (3 reports), Helen Keller International (HKI) (1 report), WHO (1 report), United Nations Office for the Coordination of Humanitarian Affairs (OCHA) (1 report), and Alliance for International Medical Action (ALIMA) (1 report).

**Table 1:** Summary of included studies in the review.

Study (country)	Population	Highlights	Type of international support	Study design/Objective
Devi 2020 (20) (Ethiopia)	100,000 refugees	Many refugees suffered from diarrhea and respiratory diseases and lacked access to medicines to treat HIV and tuberculosis.	MSF provided medical care, health promotion services, mental health care, and nutritional screening to refugees. ICRC ambulances were able to transport the wounded to the medical center.	A Report/Evaluation of humanitarian assistance to refugees in the Tigray conflict zone.
Kotsadam 2019 (21) (30 sub-Saharan African countries including countries affected by armed conflict in 2015: Niger, Mali, Burkina Faso, Democratic Republic of Congo, Central African Republic, Nigeria, South Sudan, Kenya, Somalia and Mozambique)	Women aged 12 to 45	Living in conflict zones increases pregnancy-related deaths by 2.8 per 100,000 women, with each additional conflict increasing the risk of maternal mortality by 14%.	Study funded by the Norwegian Research Council.	A micro-level analysis/To study whether local exposure to armed conflict has an impact on maternal mortality.
Harvey 2013 (22) (Sudan)	Non-applicable	Non-applicable	The World Bank and other international financial organizations are increasingly involved in disaster recovery.	A desk review/reports on the actions of international humanitarian organizations and governments in conflict zones in terms of challenges, commitments, and opportunities.
Chol 2018 (2) (Angola, Eritrea, Ethiopia, Mozambique and Rwanda)	Non-applicable	Reduction of the maternal mortality ratio	(1) Decentralization of the health system (in relation to the WHO Health System Management and Leadership Block); (2) innovations related to the WHO Health Staff Block, such as the training of community health workers (related to the WHO Health Personnel Block); (3) health financing (in relation to the funding block of the WHO health system).	A literature review/To understand best practices in the health systems of five countries in sub-Saharan Africa that experienced war between 1990 and 2015 but managed to reduce maternal mortality by more than 50% during this period.
Howell 2019 (23)(Nigeria)	Children in conflict zones	Increase in urban and rural deaths in conflict zones.	Assistance in obtaining data on the use of databases on demographic and health research and on social conflicts in Africa. The Urbana Institute and the Centre for Global Development provided internal funding for the study.	Literature review and empirical methods/This study aims to document the impact of conflict on child malnutrition in Nigeria using improved empirical methods and to complement existing literature.
Jordans 2013 (24) (Burundi and Sudan)	Children in conflict zones	community-based psychosocial and mental health care	The program was implemented through a number of partner organizations (HealthNet TPO, Burundi, and Sudan).	A case study/To describe psychosocial and mental health problems and the availability of evidence-based interventions in settings of armed conflict.
Andersen 2022 (25) (A Democratic Republic of the Congo, Mali, and Nigeria)	Victims of the violence committed by weapon	Receiving community-level mental health and psychosocial support (MHPSS) is associated with increased wellbeing among the vast majority of beneficiaries	International Committee of the Red Cross (ICRC) has offered humanitarian protection and assistance to victims of armed conflict and other situations of violence. Mental health and psychosocial support (MHPSS)	A cohort study/To explore whether this type of program correlates, as intended, with reduced psychological distress and increased daily functioning in armed conflict zones
Lokuge 2013 (26) (Democratic Republic of Congo)	children and adolescents. In conflict zones	Brief trauma-focused therapy, the current MSF mental health therapeutic intervention, appears to be effective in reducing symptoms arising from the experience of trauma.	MSF provided mental health and psychosocial support for communities affected by conflict	Retrospective study/The provision and targeting of appropriate services require a better understanding of the characteristics and requirements of children and adolescents exposed to armed conflict.
Du Mortier 2016 (27) (Kenya, Ethiopia, Uganda, Democratic Republic of the Congo and Burundi, and Zimbabwe)	People staying in conflict zones	Within its social responsibility framework, the ICRC has shown the importance and feasibility of a workplace HIV program in conflict zones.	Interventions included annual voluntary counseling and testing (VCT) uptake and direct annual costs covering awareness, testing, and antiretroviral therapy conducted by ICRC.	A retrospective analysis/To assess VCT and ART services in conflict zones
Tyndall 2020 (28) (Nigeria)	Interviews were conducted with more than 60 respondents from government, United Nations agencies, and national and international non-governmental organizations	Overall, indicators of low reproductive, maternal, neonatal, and child health (RMNCH) status and intervention coverage were found in the pre-intervention period (prior to 2016), and important improvements were noted following the arrival of international humanitarian assistance, even while armed conflict and adverse conditions persisted	Health sector intervention by the international community, including Reproductive health, Maternal health, Child health, and Nutrition (OCHA, WHO, UNICEF, UNHCR, UNFPA, NGO)	A mixed-methods case study/ To assess sexual, reproductive, maternal, newborn, child, and adolescent health and nutrition in 10 countries affected by large-scale armed conflict

**Table 2:** Summary of included documents and/or reports in the review.

Number	Study ID	Organizations	Reports	Highlights
1	2017 (29)	Ministry of Public Health	Mapping of stakeholders and interventions in support of the health sector in Niger	Improving interventions for all major endemic-epidemic diseases, including HIV/AIDS, tuberculosis, malaria, and vaccine-preventable diseases. Improving the provision of public health services by investing in strengthening data management and the health information system, the cold chain and supplies, the logistics system of medicines and laboratory inputs, targeted training for specific categories of staff, and programmatic and financial monitoring and evaluation.
2	2013 (30)	ICRC (International Committee of the Red Cross)	Annual report	ICRC provided hot meals, health care, and lodging. People injured in an attack on a military base in Agadez received critical care at the regional hospital, delivered medical supplies, and the assistance of a flown-in Health Ministry surgeon.
3	2017 (31)	Médecins Sans Frontières (MSF)	International Activity Report, Niger	MSF worked to reduce child mortality, notably during the seasonal peak of hunger and malaria, as well as to care for refugees and displaced people while also combating infections like hepatitis E.
4	2015 (32)	Save the Children	Annual Report 2015, Niger	Assistance with the treatment of malnourished children and pregnant or lactating women. Volunteer training and support for malnutrition awareness, prevention, and identification. Advocacy initiatives to include malnutrition control within public health goals and the national budget. Support for the provision of high-quality prenatal care, births, and postnatal care. Raising awareness among village communities regarding the prevention of childhood and maternal illnesses. Support for health structures that promote free access to health care. Medical agent training. Advocacy activity to enhance the proportion of the national budget allocated to health. Support for the referral system for chronically malnourished children with medical issues and women facing birthing complications.
5	2018 (33)	Action Against Hunger (ACF)	Synthesis report: Assessing the resilience of health systems to health and nutrition emergencies: Case studies from Mali and Niger	Twelve most common pathologies have been identified in Niger, including malaria, acute malnutrition, acute respiratory infections, diarrhea, urinary infections, measles, HIV, dermatoses, meningitis, cholera, whooping cough, hepatitis E, acute otitis, and gastric ulcer.
6	2018 (34)	Action Against Hunger (ACF)	Sahel: for a new approach that ensures strong and resilient health systems	ACF worked to reduce the most common pathologies identified in Niger
7	2020 (35)	Helen Keller International (HKI)	Annual report	Plumpy’Nut® dietary supplements were distributed to youngsters suffering from severe malnutrition to save their lives. Trachoma screening and surgical procedures for persons with trichiasis.
8	2020 (36)	Save the Children	Transition of Adolescents in West Africa (ATWA)	Communities show their support for teenage sexual and reproductive health services and life skills education. Adolescent sexual and reproductive health services have been improved in health facilities. Healthcare practitioners have the information, abilities, and attitudes necessary to deliver adolescent-friendly sexual and reproductive health treatments.
9	2019 (37)	Médecins Sans Frontières (MSF)	International Activity Report	Coping with the annual peak in malnutrition and malaria. MSF also treated children admitted to pediatrics at Madarounfa Hospital and the Intensive Therapeutic Nutrition Center. MSF supported health authorities by providing vaccinations, epidemiological surveillance, and emergency interventions to deal with epidemics and other emergencies. Sahel Mobile Emergency Team provided assistance to displaced people, refugees, and vulnerable local communities in conflict zones in the Tillabéri and Diffa regions.
10	2018 (38)	Médecins Sans Frontières (MSF)	International Activity Report	Outpatient consultations, malaria treatment, and hospitalization of a large number of children.
11	2020 (39)	World Health Organization (WHO)	Annual Report, Niger	8 out of 10 children were fully vaccinated before their first birthday. Out of an annual target of 967,726 children, 97% received three doses of the pentavalent vaccine and the first dose of the anti-measles vaccine. In six high-risk malaria areas, about 14 million people have benefited from long-lasting insecticide-treated mosquito nets. More than 4 million children aged 3 to 59 months have benefited from chemoprevention medication for seasonal malaria. In 2020, the improved technical platform of the Issaka Gazobi maternity unit provided better care for 4,748 pregnant women and 5,910 infants. Creating a strategy to battle HIV in critical populations. Out of the 237 existing one-stop shops for the treatment of TB/HIV co-infection have been established. Training for 314 health workers in integrated disease surveillance and response.
12	2022 (40)	United Nations Office for the Coordination of Humanitarian Affairs (OCHA)	Situation Report, Niger	Save the Children International assisted Torodi with urgent child protection needs, mental health, and delivery of protective kits. It also assisted in the water, hygiene, and sanitation sectors.
13	2020 (18)	Alliance for International Medical Action (ALIMA)	Annual report	ALIMA collaborated with its national partner BEFEN (Well-being of Women and Children in Niger) in Niger on a variety of issues, including maternity health, malnutrition, pediatrics, emergency response, malaria, and research. ALIMA and BEFEN cared for 30,292 children suffering from acute malnutrition in 2020, as well as malaria projects in Mirriah and Dakoro. ALIMA and BEFEN also carried on the “PB-mothers” project in the Maradi and Mirriah regions.
14	2020 (41)	Action Against Hunger (ACF)	Annual report	ACF was involved in multi-sectoral and health fast response mechanisms in the Maradi, Tahoua, and Diffa regions, where displaced people have critical needs. We provided medicines and emergency mobile clinics, shelter and non-food item packs, water trucking, water point rehabilitation, and disinfection services.

### 3.2 Included studies synthesis

Refugees, women, children, adolescents, victims of violence, people residing in crisis zones, and international organizations were among those who were included. There were three cohort studies, one mixed-methods case study, one case study, two literature reviews, one desk review, and one report among the included studies.

Among the seven studies reporting international health support, our findings revealed that MSF offered medical care, health promotion services, mental healthcare, nutritional screening to refugees, and mental health and psychosocial support (26, 42). Three studies revealed that ICRC ambulances were able to transport the injured to the medical center, provided interventions included annual voluntary counseling and testing (VCT) uptake and direct annual costs covering awareness, testing, and antiretroviral therapy, offered humanitarian protection and assistance to victims of armed conflict and other situations of violence, mental health, and psychosocial support (20, 25, 27). Other international organisms were involved in health sector intervention, including reproductive health, maternal health, child health, and nutrition (OCHA, WHO, UNICEF, UNHCR, UNFPA, and NGO). The most common diseases for which interventions were used included gastroenteritis, respiratory disorders, HIV, mental and psychological diseases, tuberculosis, and maternal and infantile mortality (20, 21, 23, 25, 27). According to Howell et al. (23), children in conflict zones have an increase in urban and rural fatalities (23).

In the same line, Kotsadam et al. showed that residing in war zones increased pregnancy-related deaths by 2.8 per 100,000 women, with each additional conflict raising the risk of maternal mortality by 14% (21). Furthermore, the two studies included post-conflict findings such as the decentralization of the health system (in relation to the WHO Health System Management and Leadership Block), innovations related to the WHO Health Staff Block, such as the training of community health workers (in relation to the WHO Health Personnel Block) and health financing (in relation to the WHO Health System Funding Block), reducing the maternal mortality ratio (2). Furthermore, the World Bank and other international financial institutions became more active in war rehabilitation. Other international organizations were crucial in sponsoring and supporting studies relating to health change in conflict zones. This includes support in getting data for demographic and health research databases from the Urbana Institute, the Center for Global Development, and the Norwegian Research Council.

### 3.3 Included reports synthesis

#### 3.3.1 Niger’s main conflict zones

This study identified three main areas of conflict. These are the tri-border areas of Mali, Burkina Faso, and Niger, as well as the Far East and Center-South of Niger. In addition to these conflict zones, other regions have strong security challenges, such as the Nigerien part of Liptako Gourma, the Lake Chad basin, and the south-west of the Maradi region (border areas with Nigeria). These conflict zones are significantly concentrated in three major regions of Niger: Tillabéry, Diffa, and Maradi. These conflicts are generally fueled by armed terrorist groups such as Al-Quayda in the Islamic Maghreb (AQIM), the support group for Islam and Muslims in the three-border area, and Boko Haram or the Islamic State in the far east of Niger and the Lake Chad basin. However, the three border areas seem to be the most affected by the conflicts.

#### 3.3.2 NGOs and organizations intervening in the transformation of health in conflict zones and financial support from TFPs

According to a final report in 2017 (29) on the mapping of stakeholders and interventions for HSS in support of the health sector in Niger, several partners were identified (Table 3). Twenty-three in number, these international cooperation partners operate in several health fields. Despite the heterogeneity of interventions, there is often an overlap of interventions between the different cooperation organizations (11, 43–46).

**Table 3:** Presentation of health stakeholders in Niger between 2017 and 2021.

N	Partners	Areas of intervention
1	WHO	Public health
2	AFD	Financing health through free healthcare
3	AECID	Health and Nutrition
4	UNICEF	Health Nutrition, WASH, Social Protection, and Education
5	GAVI	Immunization (Vaccines), Health Systems Strengthening, and Cold Chain Equipment Optimization.
6	United Nations Population Fund (UNFPA)	Maternal and newborn health, Sexual and reproductive health of young people and adolescents, Sexually Transmitted Infections (STIs)/ human immunodeficiency virus (HIV)/ acquired immunodeficiency syndrome (AIDS), Comprehensive sexuality Education, Family Planning, Gender-Based Violence, and Humanitarian Emergency Response.
7	BM	Reproductive Health. Nutrition Improving supply, and demand for NMRS services
8	United States Agency for International Development (USAID)	Health and Nutrition
9	Global Fund	Response to HIV, malaria, tuberculosis, and health systems strengthening
10	European Union (EU)	Free healthcare; family planning; nutrition; and support to the health system in nomadic areas
11	Humanitarian Aid and Civil Protection Department of the European Commission (ECHO)	Nutrition and Health
12	German Financial Cooperation (KfW)	Infrastructure (construction/rehabilitation, acquisition and maintenance of quality health products, and improvement of the availability of quality health products), provision of care (reproductive health and quality assurance of health services and care) of health services.
13	Belgian Technical Cooperation (BTC)	Institutional support
14	Helen Keller International (HKI)	Health, Nutrition and Water, Hygiene and Sanitation (WASH): Control of Neglected Tropical Diseases; Eye health: Fight against trichiasis, a complication of Trachoma; Community-Based Nutrition/Prevention and Malnutrition Control Program; Food Development Assistance Program: Mercy Corps/Helen Keller International Consortium.
15	Doctor of the World/ France	Santé and Nutrition
16	Concern Worldwide	Santé and Nutrition
17	Blind	Eye health, mental health, physical rehabilitation (surgery and plastered correction), early prevention of childhood disability
18	Population Services International (PSI)	Reproductive Health (Family Planning and Postabortion Care), HIV/AIDS, Women’s Empowerment and Leadership, Gender and Development
19	PATHFINDER	Reproductive health; Adolescent and youth sexual and reproductive health and resilience integrating family planning
20	Action Damien Niger	Support to the National TB Control Program
21	Nigerien Social Marketing Association (ANIMAS-SUTURA)	Sexual and reproductive health
22	Peace Movement	Promotion and Right to Health Malnutrition Hygiene and Sanitation
23	Solthis	Capacity building of national actors of local actors to enable them to fulfill their role autonomously and sustainably, Operational research, Advocacy to promote universal access to health and Training
24	Médecin sans frontière	Health and Nutrition, Vaccine response, Operations research, Infrastructure construction, Human Resources Support, and Training

**Figure 2:**
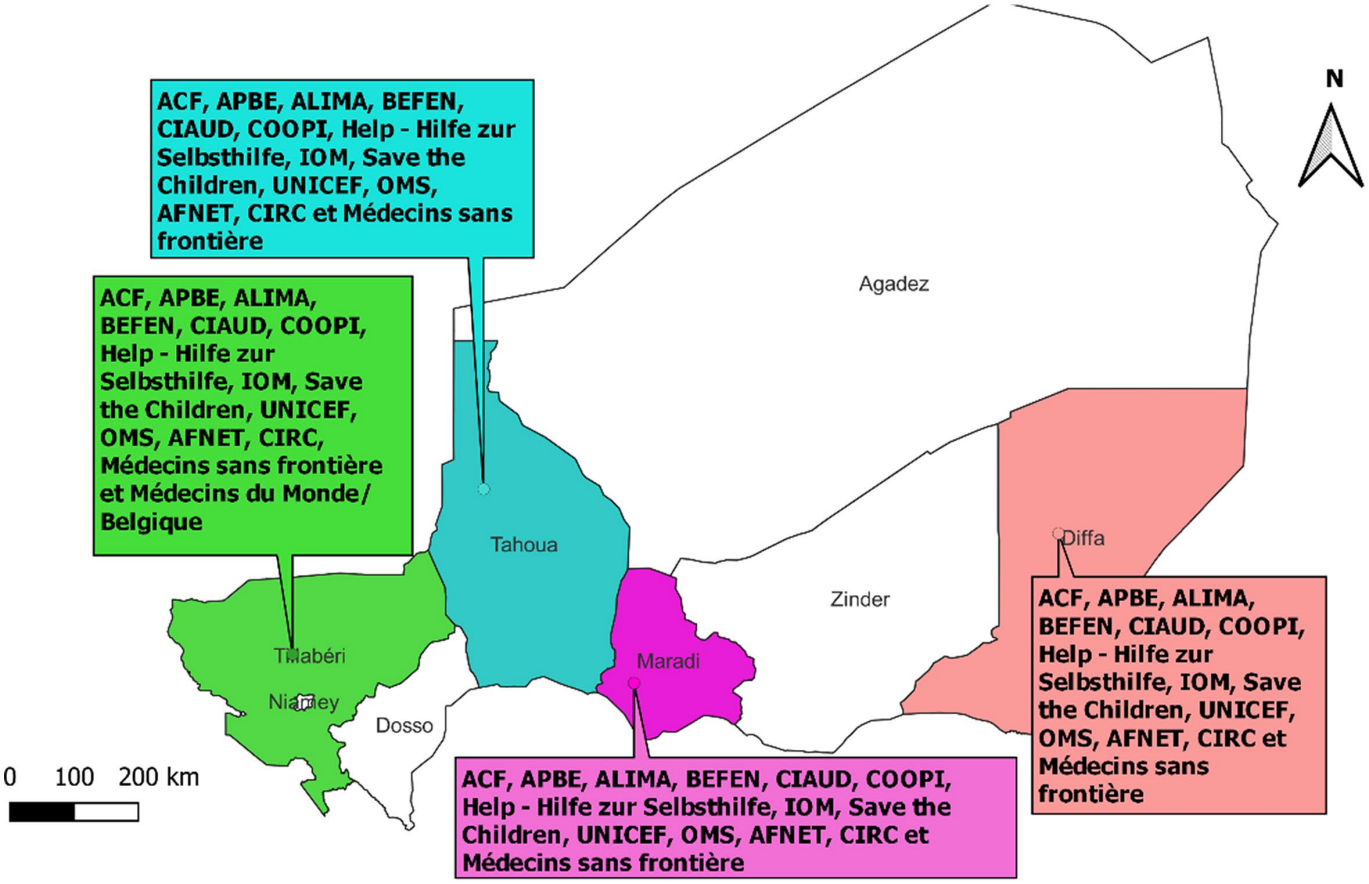
Partners involved in health in conflict zones in Niger (2022).

Table 3 and Figure 2 summarize the various partners identified in the region facing major security challenges. A total of 14 international cooperation partners working in the field of health support the Government of Niger in its public health policy. There are many donors, and the main ones identified are Médecin du Monde Belgium, Save the Children, Help, MSF, and UNICEF (Table 4).

**Table 4:** Presentations of health partners in conflict zones in Niger (2022).

Number	Partners	Funders	Health support regions
1	Action Against Hunger (ACF)	Action Against Hunger (ACF)	Maradi, Tillabéry, Tahoua and Diffa
2	Action for wellbeing (APBE)	UNFPA funding	Maradi, Tillabéry, Tahoua and Diffa
3	Alliance for International Medical Action (ALIMA) and Women’s and children’s well-being in Niger (BEFEN)	Delegation of the European Union	Maradi, Tillabéry, Tahoua and Diffa
4	International Committee for Emergency Aid and Development (CIAUD)	European Commission humanitarian aid (ECHO)	Maradi, Tillabéry, Tahoua and Diffa
5	Cooperazione Internazionale (COOPI)	Coopération internationale	Maradi, Tillabéry, Tahoua and Diffa
6	Help – Hilfe zur Selbsthilfe	AA (Department of Foreign Affairs), ECHO (Humanitarian Aid and Civil Protection)	Tillabéry, Tahoua and Diffa
7	International Organization for Migration (IOM)	United Nations	Maradi, Tillabéry, Tahoua and Diffa
8	Doctors of the World/ Belgium	European Union and the Council of Europe.	Tillabéry and Agadez
9	Save the Children	Direct and indirect funding of the U.S. government, individuals, other multilateral funding institutions, corporations, foundations, the United Nations, and other multilateral funding institutions	Maradi, Tillabéry, Tahoua and Diffa
10	United Nations Children’s Fund (UNICEF)	Nations Unies	Maradi, Tillabéry, Tahoua and Diffa
11	World Health Organization (WHO)	Nations Unies	Maradi, Tillabéry, Tahoua and Diffa
12	BEFEN/African Field Epidemiology Network (AFNET)	Department of Public Health, Population and Human Services, US Centers for Disease Control and Prevention (CDC) et AFENET	Maradi, Tillabéry, Tahoua and Diffa
13	International Committee of the Red Cross (ICRC)	International Committee of the Red Cross (ICRC)	Maradi, Tillabéry, Tahoua and Diffa
14	Doctors Without Borders (France, Switzerland, Spain, etc.)	Resources are largely financed by the private sector.	Maradi, Tillabéry, Tahoua, Diffa and Agadez

#### 3.3.3 Support for health transformation in conflict zones

This section summarizes interventions that have had an impact on the health of populations or the health system in Niger. Our research has shown that international cooperation, through the various partners listed in Table 3, has played an active role in improving the health of populations through innovative interventions or by strengthening existing capacities. Support for this transformation has materialized through the establishment of mobile clinics: mobile teams capable of providing the necessary support to affected health structures and/or, where appropriate, directly to communities through mobile services.

In addition, partners have recently responded to epidemic outbreaks through the vaccination response and the strengthening of the routine Expanded Program on Immunization (EPI). An example of this is the vaccination campaign carried out by Médecins Sans Frontières (MSF) in the department of Gouré in response to the diphtheria epidemic. In some cases, this response concerned the care of patients, as was the case in Madarounfa, an area of great instability for cholera between 2020 and 2022. The care of patients also concerned the populations of Diffa. Another support for health transformation on which several partners have focused is the training of community health personnel in managing certain nutritional or medical emergencies, including mental health. This training support has improved the quality of care for vulnerable populations in conflict-affected areas. The supply of medical equipment and drugs allowed adequate care in a context generally marked by shortages of inputs (drugs, etc.). Free healthcare for internally displaced populations (IDPs) has facilitated access to medical care for these populations. This had a positive impact on attendance at health centers and enabled adequate patient care. In addition, these interventions have enabled displaced and host populations to benefit from specialized, holistic, free, and quality health and nutrition care. An important innovation in these conflict zones has been the setting up and equipping of mobile clinics carried out by several partners with the support of the Ministry of Health, Population, and Social Action of Niger.

All of this support has resulted in improved mental health for populations living in conflict zones, increased access to care for vulnerable groups, a reduction in overall maternal mortality from 520 per 100,000 live births in 2015 to 509 maternal deaths per 100,000 live births in 2017, and improved reproductive health indicators (10). It should be noted that this decline is global throughout Niger and is not specific only to areas with strong security challenges. Still, according to the 2021 WHO report, the rate of births attended by skilled health personnel increased to 39.3% in 2021 compared to 38.8% the previous year (10, 29). The antenatal care 4 (ANC4) rate increased from 31.3% in 2020 to 34% in 2021, and postnatal consultations increased from 12.4% in 2020 to 13.9% in 2021 (10). We did not note any particular increase in these indicators in conflict zones.

An indirect approach by “scoring” (number of crosses) allowed us to identify the areas receiving the most investment. This “subjective” approach showed a classification of the pillars of the health system in which the partners invested themselves. These pillars included governance, financing, infrastructure, equipment and supplies, human resources, service delivery, and health information systems. In relation to the 6 axes of the health system, in “chronological” order, we have noted the following:

*1st: Axis 2: Improving supply, quality of care, and demand for services*

We have split this axis into two levels: general service and specific service. For the general service offer, our summary analyses of the various reports consulted (6 in total) show the use of mobile clinics in conflict zones to deal with the health problems of populations (9, 18, 35–37, 40, 47). Our results indicate that NGOs such as MSF, Alima, Save the Children, ACF, and many others have deployed mobile clinics as part of their activities, well beyond the activities they operate in traditional health centers. The establishment of 11 mobile clinics has made it possible to strengthen access to healthcare by taking care of 112,360 people in the areas of intervention of the NGO ALIMA. Training mothers in the early detection of malnutrition improved the quality of care, guaranteeing rapid treatment of children in the Tillabéry region (48).

For the specific services offered, there are those related to the nutritional care of children and those related to the health of adolescents and young children. International cooperation through NGOs and humanitarian organizations has supported the rehabilitation of nutritional recovery centers in Niger and specifically in conflict zones.

With regard to adolescent and early childhood health, we noted that international cooperation is fully committed to providing assistance to this segment of the population. In the 2020 WHO Niger annual report, we noted that this organization provided support to the Ministry of Health by training 40 trainers on the orientation program for healthcare providers for adolescents and young children. In addition, other NGOs, such as Save the Children, have developed health interventions targeting adolescents and young people, particularly in the area of sexual and reproductive health in Tillabéry. The most striking example is the training of mother educators (138 members were trained on menstrual hygiene management in schools) (36).

*2nd Axis 3: Development of human resources for health*

For this axis, our results showed that international cooperation intervenes in developing human resources in health in Diffa, Tillabéry, and Maradi. This support has taken the form of building the capacity of existing health personnel while also increasing the number of personnel to deal with health emergencies in these conflict zones. This is the case of the WHO, which is involved in improving the training of paramedical health workers, but also through its support for rural pipeline projects in the health sector, the purpose of which is to retain young people in the region (39). There is also support from partners in the field and the intervention of WHO resource persons to train rapid response teams and community health workers on prevention and care strategies. NGOs such as MSF, HKI, MdM Belgium, ACF, and ALIMA have invested heavily in building the capacity of health workers in their respective fields in conflict zones in Niger (10, 18, 48–52). On the other hand, human resource fields have remained relatively less targeted in conflict zones in Niger.

*3rd Axis 1: Improving governance and leadership*

For this last axis, the improvement of governance and leadership was noted in several activity reports of certain international cooperation partners. These are mainly the WHO, UNICEF, GAVI, USAID, BM, HKI, and ECHO, to name a few. The purpose of this improvement in governance and leadership was mostly to provide support to the country in building national capacities for better coordination in matters of leadership and governance and to actively contribute to the planning, monitoring, and evaluating funded activities.

**Figure 3:**
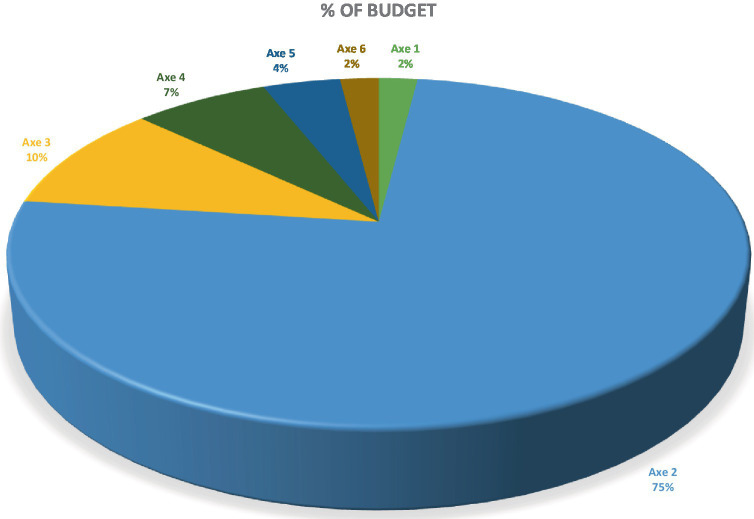
Budget breakdown of the PDS 2017–2021 according to the strategic axes.

**Figure 4:**
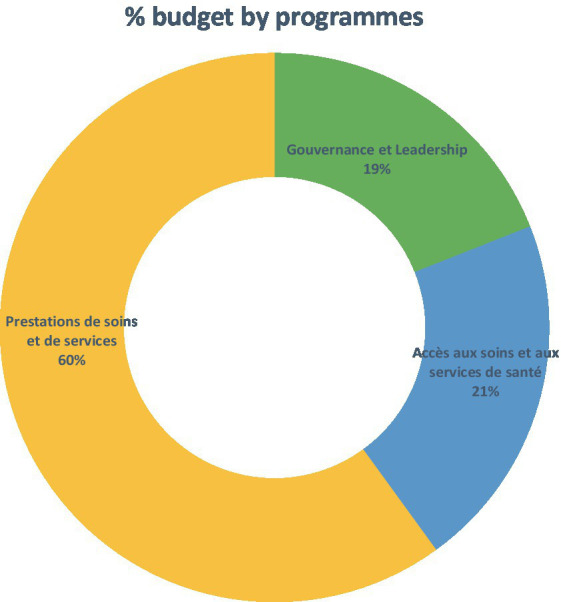
Budget breakdown of the PDS 2017–2021 according to the three programs selected.

Similarly, the assessment of the involvement of partners by the program showed that the greatest number of partners were concentrated in programs related to healthcare and services. As shown in the figures below, this information is in line with that presented in the form of the proportions of the PDS 2017–2021 budget allocated to activities related to the provision of care and services (60% compared to 19 and 21% for the two other programs, governance/leadership and access to healthcare and health services, respectively) (Figures 3, 4) (53).

#### 3.3.4 Interactions between Niger Ministry of Health and international stakeholders

In this section, the study described different interactions or links between the Niger Ministry of Health and its technical and financial partners in the process of financing and implementing projects in support of health transformation (9, 54–56). The main multilateral donors, such as the WHO, UNICEF, UNFPA, the World Bank, USAID, the European Union, ECHO, the Global Fund, and GAVI, often intervene in the fight against major endemo-epidemic diseases (emerging and re-emerging), maternal, neonatal, infant, and child health, adolescents and young people, HIV/AIDS, tuberculosis, and malaria, giving priority to prevention through vaccination and other preventive measures. They are also involved in providing public health services by contributing to data management, strengthening human resource capacities, the health information system, and health logistics (drugs and laboratory products). Bilateral donors also provide support for institutional development (e.g., CTB through training in epidemiology for health workers in the districts it supports); AFD for free healthcare; KfW for infrastructure, etc. International and/or national NGOs also work to strengthen the health system. Other national or international charitable organizations (e.g., Bill and Melinda Gates) (43, 54, 56, 57). Description of the implementation of international aid in Niger.

The Ministry of Health of Niger has a structured framework bringing together the main technical and financial partners, has a leader, and operates according to formalized mechanisms. In addition, there is a Department of Health Cooperation, which liaises between the Ministry of Health and groups of TFPs (Department of Health Cooperation). The country has put in place institutional support tools likely to contribute to improving the governance and management of health development plans. DEP/MSP is a common support fund for the implementation of the Health Development Plan (FC/PDS) based on the SWAP approach in Niger. Some financial partners have committed to channeling part of their financial assistance through this framework, which centralizes funding and facilitates the management and reallocation of funds according to country priorities. This contributes to better country ownership—alignment with Niger’s priorities, strategies, and procedures. Furthermore, structuring commitment to results-based management reinforces mutual accountability between the state and TFPs. However, this way of implementing activities is not linear and has shortcomings. For example, coordination between TFPs and government institutions is not optimized. Most financial partners use their respective tools, formats, and budget matrices instead, despite the availability of a Medium-Term Sector Expenditure Framework (CDSMT). As a result, only the large budgetary masses can sometimes be accessible, while disaggregated budgets are not. This does not allow for in-depth analysis by domain. There is also the multiplicity of channels and intermediaries; donors sometimes use several intermediaries and operators in the field (e.g., UNICEF and NGOs). Most operators in the field (NGO providers in the Regions/Districts) receive funding from several sources and do not have cost accounting.

### 3.4 Summary of the results

As a summary of the results, we found that some health transformation interventions and axes may play a crucial role in achieving universal health coverage in conflict zones according to 10 included articles and 14 reports. The interventions mainly focused on health services coverage, population coverage, quality of healthcare, and financing protection. The three axes included the following: (1) Improving supply, quality of care, and demand for services; (2) Developing human resources for health; and (3) Improving governance and leadership. The main health services coverage supports included: (1) health promotion, preventive, curative, rehabilitative, ambulance services, and emergency interventions to deal with epidemics; (2) Improving hospital and health centers services; (3) Community health services and primary healthcare; and (4) Providing healthcare to special populations (e.g., pregnant women, less than 5 children, migrants, and minors). The population coverage included all the population in the conflict zones. However, particular emphasis was placed on vulnerable populations. The quality of healthcare included general and specific services, including specialized services such as ophthalmology and orthopedics. Financing protection included collaboration between the Niger Ministry of Health and multilateral donors.

**Figure 5:**
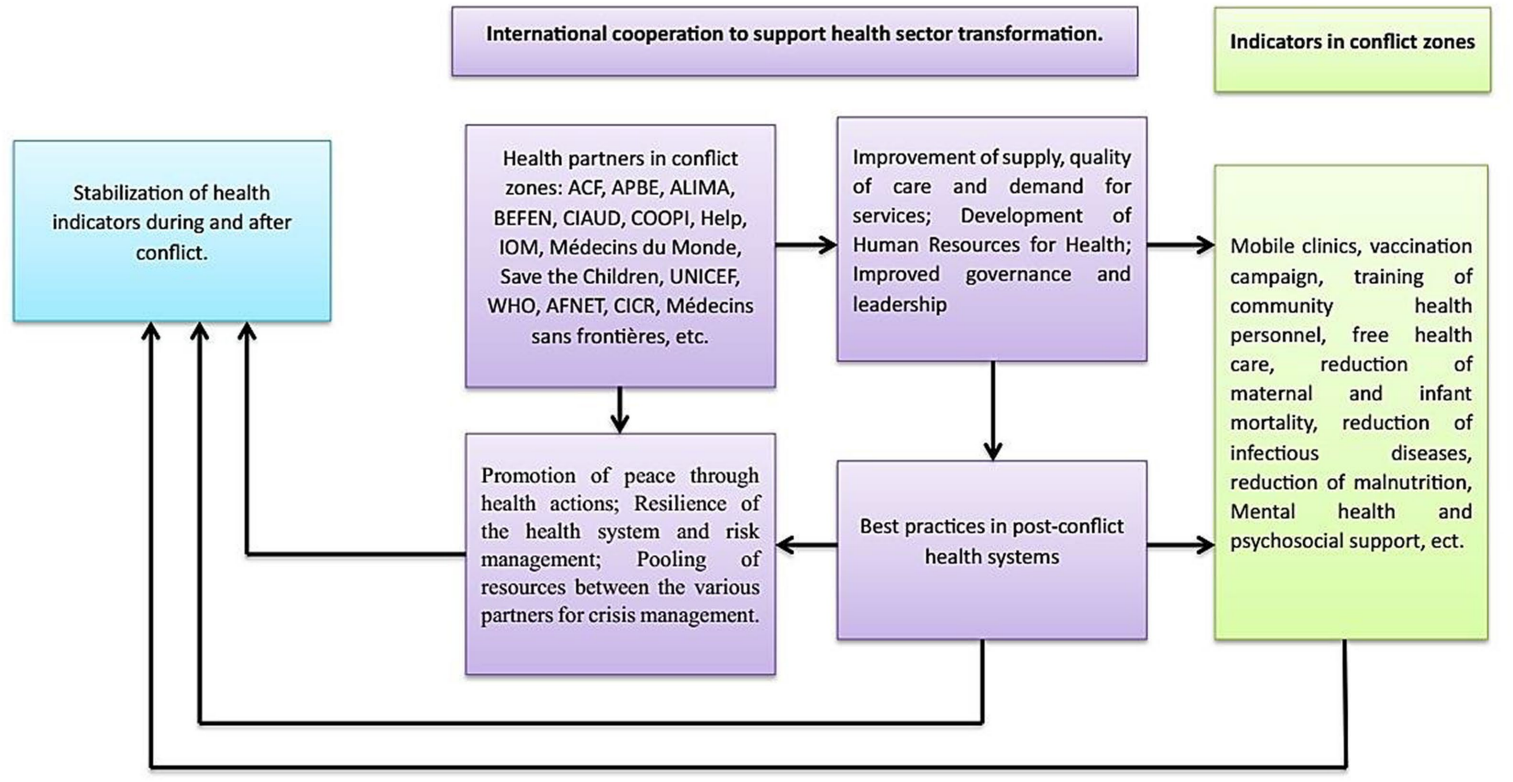
Conceptual framework: international cooperation in support of health transformation in conflict zones in Niger.

Based on our findings, we built a conceptual framework. This conceptual framework revealed that international collaboration played a substantial role in health transformation in conflict zones (Figure 5). This is shown by the increased supply of health services, quality of care and demand for services, and development of human resources for health, governance, and leadership in conflict zones. Mobile clinics, frequent immunization campaigns, community health worker training, free healthcare, reduced mother and newborn mortality, infectious diseases, malnutrition, mental health support, and critical surgical conditions, including trauma are important indicators of health in conflict zones. International cooperation was also vital in promoting peace through health actions, strengthening the health system’s resilience and risk management, and pooling resources among multiple partners for crisis management. Furthermore, best practices in health transformation in war zones were incorporated by boosting health-related research in conflict zones and increasing health-related indicators in post-conflict times. Finally, our findings demonstrated that health transformation initiatives between foreign stakeholders and the Niger Ministry of Health were well-structured.

## 4 Discussion

This review included 10 studies and 14 reports that showed evidence of international cooperation in support of health transformation in sub-Saharan conflict zones in general and in Niger in particular. The overall synthesis showed that international cooperation in support of health transformation improved the supply of health services, quality of care and demand for services, and development of human resources for health, governance, and leadership in SSA conflict zones. Mobile clinic availability, regular vaccination campaigns, training of community health personnel, free health care, reduction of maternal and infant mortality, infectious diseases, malnutrition, mental support, and essential surgical conditions, including trauma were all improved. Interventions at the peripheral level, both preventive and curative, were listed as health indicators for these interventions. These interventions have made it possible to develop several strategies, including health system resilience and risk management, development of mobile clinics, community approaches, and involvement in the management of public health problems. Our overall findings were in line with support for health transformation in conflict zones in Syria, Afghanistan, Colombia, Yemen, Iraq, Ukraine, and Palestine (58–63). A study compiled the data available from the Polish Ministry of Health, other governmental and non-governmental organizations, foundations, and medical societies, highlighting the importance of international cooperation on all activities and types of medical resources provided (64). In fact, international collaboration enabled the provision of a variety of healthcare treatments to refugees escaping Russia’s invasion of Ukraine. This included: (1) Special medical transport (trains, ambulances, and air transport) for the injured/wounded because of war actions, young patients with cancer, patients in palliative care, pregnant women, women with toddlers, and children and/or orphans from Ukraine; (2) Organize oncological treatment for young Ukrainian cancer patients; (3) Provide targeted medical assistance, including oncology, hematology, nephrology, diabetes, and neurology, at Polish clinics; (4) Establish a 24-h crisis hotline for border services and reception points accepting refugees from Ukraine to assist disabled individuals (64, 65); (5) Proxy of the Minister of Health for transfer and ongoing treatment outside the Republic of Poland for Ukrainian patients staying on the territory of the Republic of Poland; (6) Launch of the Teleplatform of First Contact with permanent medical services supplied to Ukrainians (64, 66); (7) The establishment of a free helpline in Ukraine for those in need of psychological support (64, 67); (8) Launch of a free search engine for medical entities that allows communication in Ukrainian, Russian, or English language; and (9) Glossaries and medical translators (64). Along the same line, the ICC began examining the aforementioned crimes perpetrated on Ukrainian soil during the continuing Russian–Ukrainian war (68). These include both acts of violence against the life and health of civilians and combatants (wilful killing, torture or inhuman treatment, serious injury to the body or health, physical mutilation, and medical or scientific experiments that cause death or seriously endanger the health) and acts of sexual violence against the right to self-determination (68). The most important fact is that the ICC has been using forensic medicine in addressing health-related war crimes (68). The wars in Gaza have led to the immediate and evident destruction of healthcare facilities (69, 70), including hospitals, clinics, and medical supply centers, often targeted directly or damaged as collateral. This has left the fragile healthcare system in disarray, reducing its ability to provide care. A study reported that international cooperation and humanitarian aid are essential to rebuilding healthcare infrastructure, ensuring the availability of medical supplies, and supporting mental health services in Gaza (69).

Based on the positive impact of this evidence on health transformation in conflict zones, particular emphasis was placed on Niger, where armed conflicts are still ongoing. Our review showed that international support for health transformation in conflict zones in Niger is focused on three axes, mainly support for health transformation in conflict zones, including improving supply, quality of care, and demand for services, and development of human resources for health; and improving governance and leadership. In Niger, many humanitarian actors intervene in an integrated way in the logic of strengthening the national system of existing community-based epidemiological surveillance, in particular through the involvement of a large network of community relays in the surveillance and vaccination system covering almost all the villages of intervention of the project on the one hand and by building the capacities of community actors in charge of surveillance and those of the Ministry of Health on the other hand (52, 54, 55, 71, 72). Vaccination strategies have been developed by the actors either in the camps or through mobile teams, but also through the management of childhood illnesses, mental illnesses, and the promotion of sexual and reproductive health (12, 20, 22, 46, 73–75). Strengthening health systems and integrating the management of acute malnutrition into health systems, with the support of national governments and international organizations, are priorities (76). It has been mentioned that one of the indicators of good governance is decentralization, particularly by facilitating the participation of subnational actors in decision-making (2). SSA has made progress in the field of leadership thanks to this system of decentralization supported by international cooperation (77, 78). With regard to the development of human resources, our results focused on capacity building, whereas in other countries, it was the massive recruitment and training of healthcare workers (CHWs) that were preferred to deal with the shortage of human resources in conflict zones within the framework of international cooperation support. Our review also showed that there was no study or report discussing access to abortion for women raped in armed conflicts. International cooperation should highlight this topic as the effects of conflict on abortion are more rarely incorporated into empirical research on the consequences of armed conflict (79). In view of the considerable problems inherent in the access to abortion for women raped in armed conflicts, it will be fundamental to establish pathways for people to access abortions with pills (16). A solution could also be to send doctors, gynecologists, and volunteers to properly inform victims about the risks and benefits of abortion, but also about possible alternatives (16). International cooperation should also focus on training doctors and health workers to assist women raped in armed conflicts, both gynecologically and psychologically (16). If abortion cannot be carried out in a hosted country, it will be necessary for international organizations to ensure a rapid transfer to countries where abortion is not restricted (16).

In addition, our analysis also showed that the decentralization of the health system in relation to the WHO Health System Management and Leadership Block, innovations related to the WHO Health Staff Block, such as the training of community health workers in relation to the WHO Health Personnel Block, and health financing in relation to the WHO Health System Funding Block have reduced the maternal mortality ratio by 50% in Angola, Eritrea, Ethiopia, Mozambique, and Rwanda over a long period after conflict (2). Unlike Niger, health financing in Angola is less dependent on external donors; only 14% of health financing in Angola comes from aid (80). The state contributes 80% of total health expenditure, and personal expenditure represents only 20% (81, 82). Twenty percent of revenue is generated by private health insurance through the National Insurance Company of Angola, and the government provides the rest of the public funding (6). In Ethiopia, more than 50% of healthcare financing comes from the Ethiopian government and out-of-pocket (OOP) payments (83).

In Niger, the interactions between the Ministry of Health and international stakeholders are structured. The Ministry of Health of Niger has a structured framework bringing together the main technical and financial partners, has a leader, and operates according to formalized mechanisms. Some financial partners have committed to channeling part of their financial assistance through this framework, which centralizes funding and facilitates the management and reallocation of funds according to country priorities. However, restructuring or transformative healthcare activities in conflict zones in short and long terms are needed as the WHO and world bank estimations in Niger about the service coverage index for Universal Health Coverage was 35% in 2019 compared to 33% in 2017 (38). Indeed, long-term health transformation in conflict zones must also be based on evidence-based practices. Even though long-term health transformation in conflict zones needs more studies, decentralization of the health system, such as the WHO Health System Funding Block, has shown its effectiveness in achieving health coverage. Furthermore, adequate state health expenditure associated with private insurance and international stakeholders’ funds may play an important role in achieving health coverage in conflict zones.

According to the literature, this is the first review of its genre to be conducted in both Niger and SSA. The strength of this review is that it provides ample evidence of international cooperation in support of health transformation in achieving universal health coverage in conflict zones in Niger in particular and in SSA in general. This review recommends that six pillars: 1) governance, 2) financing, 3) infrastructure, equipment and supplies, 4) human resources, 5) service delivery, and 6) health information systems, should be improved in conflict zones in the short and long terms. More studies are still needed to bring more evidence on empowering these six pillars in the long term. In addition, more research funds on supporting international cooperation for published research in conflict areas are needed. This could be explained by the scarcity of studies in this domain as few studies were found according to our results.

However, the review uncounted some weaknesses, the difficulty of accessing NGO reports, contacting the heads of NGOs to obtain certain information, ineffective updating of the data available at the MSP/PAS, including the list of NGOs, and poor completeness of certain reports provided. This review did not include the quantification of all universal health coverage indicators, such as rate of health coverage (0–5 km), rate of immunization from 12 to 23 months, rate of tetanus vaccination among women of reproductive age prenatal care, qualified persons assisting with delivery, delivery at a health facility, access to abortion for women raped in armed conflicts, and so on. Furthermore, because the first part of this study included a desk review, this does not typically include the risk of bias assessment. Finally, only one study was found to include Niger, and the rest were the reports.

## 5 Conclusion

Through this documentary analysis, it appears that international cooperation is actively involved in supporting the strengthening and transformation of the health system in conflict zones internationally, particularly in Niger. Integrating the activities of all partners in support of Niger’s health system remains a problem. This study shows that the support of these multilateral, bilateral, and specific organizations is substantial in transforming the health system in conflict zones. Successful interventions in achieving universal health coverage in conflict zones, such as health services coverage, population coverage, quality of healthcare, and financing protection, are needful. Health interventions have enabled the monitoring of indicators, the prevention of diseases through vaccination and the promotion of hygiene, and the management of essential medico-surgical conditions or those associated with conflict trauma. However, international partners and MSP/P/AS officials have to restructure or transform healthcare activities in conflict zones in short and low terms.

In addition to promoting peace through health actions, this approach fits perfectly with the perspectives of WHO through “The Global Health for Peace Initiative,” which aims to strengthen and concretize the link between health, social cohesion, and peace, emphasizing the unique role that public health programs can play in bringing different groups together and building trust. It looks at the different components of peace, including political peace and social cohesion at the community level.

## Data availability statement

The original contributions presented in the study are included in the article/supplementary materials, further inquiries can be directed to the corresponding author.

## Author contributions

MD: Data curation, Formal analysis, Investigation, Methodology, Writing – original draft, Writing – review & editing, Conceptualization, Project administration, Validation. AG: Conceptualization, Methodology, Validation, Writing – original draft, Writing – review & editing. FZ: Conceptualization, Supervision, Visualization, Writing – original draft, Writing – review & editing. BM: Conceptualization, Investigation, Writing – original draft, Writing – review & editing. EI: Methodology, Validation, Visualization, Writing – review & editing. OT: Conceptualization, Data curation, Methodology, Validation, Visualization, Writing – original draft, Writing – review & editing. BO: Conceptualization, Data curation, Formal analysis, Investigation, Methodology, Project administration, Supervision, Validation, Visualization, Writing – original draft, Writing – review & editing. MK: Data curation, Funding acquisition, Methodology, Project administration, Supervision, Validation, Writing – review & editing. GD: Investigation, Validation, Writing – review & editing. AC: Validation, Visualization, Writing – review & editing. SM: Conceptualization, Writing – review & editing. KB: Validation, Writing – review & editing. JT: Investigation, Validation, Writing – review & editing. HH: Investigation, Validation, Visualization, Writing – review & editing. HM: Investigation, Validation, Writing – review & editing. N’ZK: Investigation, Methodology, Validation, Writing – review & editing. JLT: Data curation, Formal analysis, Investigation, Methodology, Supervision, Validation, Visualization, Writing – original draft, Writing – review & editing. PK: Formal analysis, Investigation, Methodology, Supervision, Validation, Visualization, Writing – original draft, Writing – review & editing. CW: Methodology, Supervision, Validation, Writing – review & editing. B-PM: Conceptualization, Data curation, Funding acquisition, Investigation, Methodology, Project administration, Supervision, Validation, Visualization, Writing – original draft, Writing – review & editing.

## Glossary

ACF: Action Against Hunger
AECID: Spanish Agency for International Cooperation for Development
AFD: French Development Agency
AFNET: African Field Epidemiology Network
ALIMA: Alliance for International Medical Action
ANC4: antenatal care 4
ANIMAS-SUTURA: Nigerien Social Marketing Association
APBE: Action for Well-Being
AQIM: Al-Quayda in the Islamic Maghreb
ATWA: Transition of Adolescents in West Africa
BEFEN: Well-Being of Women and Children in Niger
CDSMT: Medium Term Sector Expenditure Framework
CIAUD: International Committee for Emergency Aid and Development
ICRC: International Committee of the Red Cross
COOPI: International Cooperation
BTC: Belgian Technical Cooperation
DS: Health district
DHS: demographic health surveys
ECHO: European Commission Humanitarian Aid and Civil Protection Department
EPI: Expanded Vaccination Program
PDS: Health development plan
EU: European Union
GAVI: Global Alliance for Vaccines and Immunization
GDP: Gross Domestic Product
HIV: Human immunodeficiency virus
HKI: Helen Keller International
INSO: International NGO Safety Organization
IOM: International Organization for Migration
IDPs: Internally Displaced Persons
KfW: German Financial Cooperation
GAM: Global acute malnutrition
SAM: Severe acute malnutrition
MSF: Doctors Without Borders
MSP/P/AS: Minister of Public Health
NGO: Non-Governmental Organization
NSAG: Non-state armed groups
OCHA: United Nations Office for the Coordination of Humanitarian Affairs
OOP: Out-of-pocket
PBF: Performance-based financing
PSA: POPULATION and Social Affairs
PRISMA: Planned method for reporting systematic reviews and meta-analyses
PSI: Population Services International
RSS: Strengthening the health system
SLR: Systematic literature review
UNICEF: United Nations Children’s Fund
UNDAF: United Nations Development Assistance Framework.
UNFRA: United Nations Population Fund
USAID: United States Agency for International Development
WASH: Health Nutrition and Water Hygiene and Sanitation
WFP: World Food Program
WHO: World Health Organization

## References

[1] Raleigh C. Political marginalization, climate change, and conflict in African Sahel states. Int Stud Rev. (2010) 12:69–86. doi: 10.1111/j.1468-2486.2009.00913.x

[2] Chol C Negin J Garcia-Basteiro A Gebrehiwot TG Debru B Chimpolo M . Health system reforms in five sub-Saharan African countries that experienced major armed conflicts (wars) during 1990-2015: a literature review. Glob Health Action. (2018) 11:1517931. doi: 10.1080/16549716.2018.1517931, PMID: 30270772 PMC7011843

[3] Human Security Research Group. (2013). The decline in global violence: Evidence, explanation, and contestation. Available at: https://www.ualberta.ca/~tkeating/HSR13.pdf (Accessed May 25, 2023).

[4] Themnér L Wallensteen P. Armed conflicts, 1946–2011. J Peace Res. (2012) 49:565–75. doi: 10.1177/0022343312452421

[5] Witter S Falisse J-B Bertone MP Alonso-Garbayo A Martins JS Salehi AS . State-building and human resources for health in fragile and conflict-affected states: exploring the linkages. Hum Resour Health. (2015) 13:1–15. doi: 10.1186/s12960-015-0023-5 25971407 PMC4488955

[6] Druetz T Browne L Bicaba F Mitchell MI Bicaba A. Effects of terrorist attacks on access to maternal healthcare services: a national longitudinal study in Burkina Faso. BMJ Glob Health. (2020) 5:e002879. doi: 10.1136/bmjgh-2020-002879, PMID: 32978211 PMC7520815

[7] Ramadan M Tappis H Uribe MV Brieger W. Access to primary healthcare Services in Conflict-Affected Fragile States: a subnational descriptive analysis of educational and wealth disparities in Cameroon, Democratic Republic of Congo, Mali, and Nigeria. Int J Equity Health. (2021) 20:1–12. doi: 10.1186/s12939-021-01595-z 34895244 PMC8665620

[8] Van Lerberghe W Matthews Z Achadi E Ancona C Campbell J Channon A . Country experience with strengthening of health systems and deployment of midwives in countries with high maternal mortality. Lancet. (2014) 384:1215–25. doi: 10.1016/S0140-6736(14)60919-3 24965819

[9] OCHA. (2022). Hamanitarian response. Available at: https://www.humanitarianresponse.info/fr/operations/niger/document/niger-nigerhrp2022 (Accessed May 25, 2023).

[10] CTE/PDR. (2015). Plan De Developpement Regional (PDR). Available at: https://www.migration-spccm.ne/sites/default/files/2021-06/PDR%20ZINDER.docx (Accessed May 25, 2023).

[11] UNHCR-Africa. (2022). UNHCR Global Trends 2018. Available at: https://www.unhcr.org/statistics/unhcrstats/5d08d7ee7/unhcr-global-trends-2018.html (Accessed May 25, 2023).

[12] Wagner Z Heft-Neal S Bhutta ZA Black RE Burke M Bendavid E. Armed conflict and child mortality in Africa: a geospatial analysis. Lancet. (2018) 392:857–65. doi: 10.1016/S0140-6736(18)31437-5, PMID: 30173907 PMC6338336

[13] OMS. (2017). Stratégie de Coopération de l’OMS avec le Niger – SCP 2017–2021. Available at: https://www.afro.who.int/fr/publications/strategie-de-cooperation-de-loms-avec-le-niger-scp-2017-2021 (Accessed May 25, 2023).

[14] SDS SAHEL – NIGER. (2011). Strategie de Developpement et de Securite Dans Les Zones Sahelo – Sahariennes Du Niger. Available at: https://www.ipinst.org/images/pdfs/sds_version_francaise.pdf (Accessed May 25, 2023).

[15] CICR. (2018). Réduire la violence contre les soins de santé au Niger, en République centrafricaine et au Nigéria. Available at: https://healthcareindanger.org/wp-content/uploads/2019/03/4369_001_HCID_Modifier-comportement_web.pdf (Accessed May 25, 2023).

[16] Cioffi A Cecannecchia C Cioffi F. Violation of the right to abortion at the time of the war in Ukraine. Sex Reprod Healthc. (2022) 33:100738. doi: 10.1016/j.srhc.2022.100738, PMID: 35640526

[17] OCHA. (2022). Nations Unies. Niger: une situation alimentaire et nutritionnelle toujours inquiétante. Available at: https://news.un.org/fr/story/2022/12/1130962 (Accessed May 25, 2023).

[18] ALIMA. (2022). Alima – Alliance International for Medical Actions-Hip. Available at: https://alima.ngo/en/2022-3/ (Accessed May 25, 2023).

[19] Page MJ McKenzie JE Bossuyt PM Boutron I Hoffmann TC Mulrow CD . The PRISMA 2020 statement: an updated guideline for reporting systematic reviews. Rev Esp Cardiol (Engl Ed). (2021) 74:790–9. doi: 10.1016/j.rec.2021.07.010, PMID: 34446261

[20] Devi S. Humanitarian access deal for Tigray. Lancet. (2020) 396:1871. doi: 10.1016/S0140-6736(20)32669-6, PMID: 33308456

[21] Kotsadam A Østby G. Armed conflict and maternal mortality: a micro-level analysis of sub-Saharan Africa, 1989–2013. Soc Sci Med. (2019) 239:112526. doi: 10.1016/j.socscimed.2019.112526 31520880

[22] Harvey P. International humanitarian actors and governments in areas of conflict: challenges, obligations, and opportunities. Disasters. (2013) 37:S151–70. doi: 10.1111/disa.12019 23876011

[23] Howell E Waidmann T Birdsall N Holla N Jiang K. The impact of civil conflict on infant and child malnutrition, Nigeria, 2013. Matern Child Nutr. (2020) 16:e12968. doi: 10.1111/mcn.12968, PMID: 32048455 PMC7296780

[24] Jordans MJ Tol WA Susanty D Ntamatumba P Luitel NP Komproe IH . Implementation of a mental health care package for children in areas of armed conflict: a case study from Burundi, Indonesia, Nepal, Sri Lanka, and Sudan. PLoS Med. (2013) 10:e1001371. doi: 10.1371/journal.pmed.1001371, PMID: 23335863 PMC3545867

[25] Andersen I Rossi R Hubloue I. Community-level mental health and psychosocial support during armed conflict: a cohort study from the Democratic Republic of the Congo, Mali, and Nigeria. Front Public Health. (2022) 10:815222. doi: 10.3389/fpubh.2022.815222, PMID: 35419344 PMC8995431

[26] Lokuge K Shah T Pintaldi G Thurber K Martínez-Viciana C Cristobal M . Mental health services for children exposed to armed conflict: Médecins Sans Frontières’ experience in the Democratic Republic of Congo, Iraq and the occupied Palestinian territory. Paediatrics Int Child Health. (2013) 33:259–72. doi: 10.1179/2046905513Y.0000000098, PMID: 24196701 PMC3817578

[27] Du Mortier S Mukangu S Sagna C Nyffenegger L Aebischer PS. A decade of an HIV workplace programme in armed conflict zones; a social responsibility response of the international committee of the red cross. J Occup Med Toxicol. (2016) 11:1–10. doi: 10.1186/s12995-016-0119-4 27247611 PMC4886433

[28] Tyndall JA Ndiaye K Weli C Dejene E Ume N Inyang V . The relationship between armed conflict and reproductive, maternal, newborn and child health and nutrition status and services in northeastern Nigeria: a mixed-methods case study. Confl Heal. (2020) 14:1–15. doi: 10.1186/s13031-020-00318-5 PMC7664044 33292426

[29] Florent Blaise B. (2017). Réalisation d’une cartographie des intervenants et des interventions en appui au secteur de la santé au Niger. Available at: https://extranet.who.int/sph/sites/default/files/remap/Rapport%20Niger%20%281%29.pdf (Accessed May 25, 2023).

[30] ICRC. (2013). Annual report. Available at: https://www.icrc.org/fr/doc/resources/documents/annual-report/icrc-annual-report-2013.htm (Accessed May 25, 2023).

[31] MSF. (2017). Rapport International d'activités 2017-Niger. Available at: https://www.msf.org/sites/default/files/2018-08/msf-rapport-international-activit%C3%A9s-2017.pdf (Accessed August 31, 2022).

[32] ATWA. (2021). IATI-save-the-children-ATWA-rapport-annuel-2020-Niger-Mai-2021-20210331010328.pdf. Available at: https://aidstream.org/files/documents/IATI-Save-the-Children-ATWA-rapport-annuel-2020-Niger-Mai-2021-20210331010328.pdf (Accessed August 31, 2022)

[33] ACF. (2018). Synthesis report: Assessing the resilience of health systems to health and nutrition emergencies: Case studies from Mali and Niger. Available at: https://knowledgeagainsthunger.org/technical/sahel-a-new-approach-to-guarantee-strong-and-resilient-health-s (Accessed May 25, 2023).

[34] ACF. (2018). Sahel Evaluation de la résilience des systèmes de santé face aux urgences sanitaires et nutritionnelles: Etudes de cas au Mali et au Niger. Available at: https://www.accioncontraelhambre.org/sites/default/files/documents/sahel_evaluation_de_la_resilience_des_s (Accessed May 25, 2023).

[35] Helen Keller International (HKI). (2020). Annu Rep. Available at: https://www.hki.org/wp-content/uploads/2021/08/HelenKellerIntl_RapportAnnuel2020.pdf (Accessed May 25, 2023).

[36] Save children. (2020). La Transition des Adolescents en Afrique de l’Ouest (ATWA). Available at: https://www.alignplatform.org/resources/adolescent-transition-west-africa-atwa (Accessed May 25, 2023).

[37] MSF. (2019). International Activity Report. Available at: https://www.msf.org/fr/rapport-international-dactivit%C3%A9s-2019 (Accessed May 25, 2023).

[38] MSF. (2018). International Activity Report. Available at: https://www.msf.org/sites/default/files/2019-08/msf-international-activity-report-2018.pdf (Accessed August 31, 2022).

[39] WHO. (2020). Annual Report, Niger. Available at: https://www.afro.who.int/fr/countries/niger/publication/rapport-annuel-dactivites-2021-de-loms-niger (Accessed May 25, 2023).

[40] OCHA. (2022). Situation Report, Niger. Available at: https://reports.unocha.org/en/country/niger (Accessed May 15, 2023).

[41] ACF. (2020). Annual report. Available at: https://www.actioncontrelafaim.org/wp-content/uploads/2022/12/Annual-report-2020.pdf (Accessed August 31, 2022).

[42] Devi S. Crisis in the Chad Basin. Lancet. (2018) 392:904–5. doi: 10.1016/S0140-6736(18)32250-5, PMID: 30238879

[43] OCHA. (2022). Plan de Réponse Humanitaire Niger. Available at: https://www.humanitarianresponse.info/sites/www.humanitarianresponse.info/files/documents/files/niger_hrp_2022_3.pdf (Accessed May 25, 2023).

[44] ALIMA. (2020). Annual report. Available at: https://alima.ngo/wp-content/uploads/2021/12/Rapport-annuel_ALIMA_2020.pdf (Accessed May 25, 2023).

[45] Action Contre la Faim. (2019). ACF-RMA-2019.pdf. Available at: https://www.actioncontrelafaim.org/wp-content/uploads/2020/09/RMA-2019.pdf (Accessed August 31, 2022)

[46] Rapport international d’activités 2019 | MSF. (2022). Le bilan de l'année. Available at: https://www.msf.org/fr/rapport-international-dactivit%C3%A9s-2019 (Accessed August 31, 2022)

[47] AFNET § BEFEN. (2022). Surveillance renforcée de la polio au niveau communautaire en Afrique. ONG Bienêtre de la femme et de l’enfant au Niger: 2020-2022. Unpublished report.

[48] Institut National de la Statistique. (2020). Enquete Nationale De Nutrition Selon La Methodologie Smart, Niger. Available at: https://www.stat-niger.org/wp-content/uploads/nutrition/RAPPORT_SMART_Niger_2020_VF.pdf (Accessed May 25, 2023).

[49] Amodou MI Didier L. Injuries of Boko haram insurgency in south-East Niger Republic. J West African Coll Surg. (2018) 8:22. PMC7368572 32754455

[50] United Nations. (2023). Sustainable development goals. Available at: https://www.un.org/sustainabledevelopment/

[51] Ndegwa SN. (2002). Decentralization in Africa: A stocktaking survey Africa region. Available at: https://www.istr.org/conferences/barcelona (Accessed May 25, 2023).

[52] HPC. (2023). HPC – Project Module: View Project. Available at: https://projects.hpc.tools/project/187436/view (Accessed August 16, 2022)

[53] Owoaje ET Uchendu OC Ajayi TO Cadmus EO. A review of the health problems of the internally displaced persons in Africa. Niger Postgrad Med J. (2016) 23:161–71. doi: 10.4103/1117-1936.196242, PMID: 28000636

[54] DRSP. (2015). Direction régionale de la santé publique de l’action sociale et de la population de Zinder (DRSP/AS/P).

[55] International NGO Safety Organisation (INSO). (2021). International NGO Safety organisation (INSO). Available at: https://ngosafety.org/ (Accessed August 18, 2022)

[56] Rapport projet AFNET§BEFEN. (2022). Surveillance renforcée de la polio au niveau communautaire en Afrique. ONG Bienêtre de la femme et de l’enfant au Niger 2020–2022.

[57] OCHA Niger. (2021). Liste de Contacts Humanitaires – Niger | ReliefWeb. Available at: https://reliefweb.int/report/niger/ocha-niger-liste-de-contacts-humanitaires-novembre-2021 (Accessed August 16, 2022)

[58] Hassan G Ventevogel P Jefee-Bahloul H Barkil-Oteo A Kirmayer LJ. Mental health and psychosocial wellbeing of Syrians affected by armed conflict. Epidemiol Psychiatr Sci. (2016) 25:129–41. doi: 10.1017/S2045796016000044, PMID: 26829998 PMC6998596

[59] WHO. (2021). Afghanistan Health Cluster: ensuring continuity of health services. Available at: https://healthcluster.who.int/newsroom/news/item/15-11-2021-afghanistan-health-cluster-advocating-to-ensure-continuity-of-health-services (Accessed May 25, 2023).

[60] UNHCR. (2003). Bringing health care to the people in Colombia’s conflict zone. Available at: https://www.unhcr.org/news/news/bringing-health-care-people-colombias-conflict-zone (Accessed May 25, 2023).

[61] Worldbank. (2019). Yemen Emergency Health and Nutrition Project. Available at: https://www.worldbank.org/en/news/factsheet/2019/05/14/yemen-emergency-health-and-nutrition-project (Accessed May 25, 2023).

[62] Ibrahim S Al-Dahir S Al Mulla T Lami F Hossain SM Baqui A . Resilience of health systems in conflict affected governorates of Iraq, 2014–2018. Confl Heal. (2021) 15:1–9. doi: 10.1186/s13031-021-00412-2 PMC8521490 34663395

[63] UNICEF. (2019). MHPSS Rapid Review Palestine. Available at: https://www.unicef.org/sop/media/1031/file/MHPSS%20Rapid%20Review%20Palestine%202019.pdf (Accessed May 25, 2023).

[64] Fatyga E Dzięgielewska-Gęsiak S Muc-Wierzgoń M. Organization of medical assistance in Poland for Ukrainian citizens during the Russia-Ukraine war. Front Public Health. (2022) 10:904588. doi: 10.3389/fpubh.2022.904588, PMID: 35874981 PMC9300910

[65] Poland Government. (2022). Medical assistance for Ukrainian citizens. Available at: https://www.gov.pl/web/zdrowie/pomoc-medyczna-dlaukrainy/; https://www.gov.pl/web/health/medical-help-for-Ukraine (Accessed May 25, 2023).

[66] Collegium Cavitas. (2023). Medical and psychological help. Available at: https://www.civitas.edu.pl/en/our-university/supportmeasures/medical-and-psychological-help (Accessed May 25, 2023).

[67] Instytut Matki i Dziecka. (2023). LikarPL. Available at: https://likar.mz.gov.pl/ (Accessed May 25, 2023).

[68] Cioffi A Cecannecchia C. Role of forensic medicine in addressing the war crimes: perspective from Russia-Ukraine conflict during the COVID-19 pandemic. Med Sci Law. (2023) 63:168–73. doi: 10.1177/00258024221125135, PMID: 36083178 PMC9465054

[69] Ahmed SK. Addressing the effects of war on Gaza’s healthcare system. Cureus. (2023) 15:e50036. doi: 10.7759/cureus.50036, PMID: 38186426 PMC10768328

[70] Zughbur MR. Protect civilians’ lives and health care in Gaza. Lancet. (2023) 402:1620–1. doi: 10.1016/S0140-6736(23)02402-9 39492155

[71] OCHA. (2021). Niger: Aperçu des besoins humanitaires 2021. Available at: https://www.humanitarianresponse.info/en/operations/niger/document/niger-aper%C3%A7u-des-besoins-humanitaires-201 (Accessed May 25, 2023).

[72] RAPPORT. (2020). RAPPORT_SMART_Niger_2020_VF.pdf. Available at: https://www.stat-niger.org/wp-content/uploads/nutrition/RAPPORT_SMART_Niger_2020_VF.pdf (Accessed August 31, 2022)

[73] ALIMA. (2020). Rapport-annuel_ALIMA_2020.pdf. Available at: https://alima.ngo/wp-content/uploads/2021/12/Rapport-annuel_ALIMA_2020.pdf (Accessed August 31, 2022)

[74] Rapport annuel d’activités 2021 de l’OMS Niger. (2021). OMS | Bureau régional pour l’Afrique. Available at: https://www.afro.who.int/fr/countries/niger/publication/rapport-annuel-dactivites-2021-de-loms-niger (Accessed August 31, 2022)

[75] Ilboudo P Siri A. La Politique de Gratuité des Soins de Santé Maternelle et Infantile au Burkina Faso: Effets et Pérennité de l’Intervention. (2022). Available at: https://publication.aercafricalibrary.org/items/d19d4273-6afe-44a7-adc3-a6d036bc4813 (Accessed August 31, 2022).

[76] Rapport annuel d’activités 2020 de l’OMS Niger. (2020). OMS | Bureau régional pour l’Afrique. Available at: https://www.afro.who.int/fr/publications/rapport-annuel-dactivites-2020-de-loms-niger (Accessed August 31, 2022)

[77] Reich MR Javadi D Ghaffar A. Introduction to the special issue on “effective leadership for health systems”. Health Syst Reform. (2016) 2:171–5. doi: 10.1080/23288604.2016.1223978, PMID: 31514592

[78] Manzi A Munyaneza F Mujawase F Banamwana L Sayinzoga F Thomson DR . Assessing predictors of delayed antenatal care visits in Rwanda: a secondary analysis of Rwanda demographic and health survey 2010. BMC Pregnancy Childbirth. (2014) 14:290. doi: 10.1186/1471-2393-14-290, PMID: 25163525 PMC4152595

[79] O’Brien ML. The consequences of the Tajikistani civil war for abortion and miscarriage. Popul Res Policy Rev. (2021) 40:1061–84. doi: 10.1007/s11113-020-09624-5, PMID: 34658465 PMC8513771

[80] Chepkurui V Amponsah-Dacosta E Haddison EC Kagina BM. Characterization of National Immunization Programs in the context of public health emergencies: a case study of 13 countries in the WHO Africa region. Front Public Health. (2021) 9:736532. doi: 10.3389/fpubh.2021.736532, PMID: 34650952 PMC8505981

[81] Ratnayake R Degomme O Roberts B Spiegel P. Conflict and health: seven years of advancing science in humanitarian crises. Confl Heal. (2014) 8:7. doi: 10.1186/1752-1505-8-7, PMID: 24843382 PMC4024652

[82] Antarou L Ridde V Kouanda S Queuille L. Health staff workload in a context of user fees exemption policy for health care in Burkina Faso and Niger. Bull Soc Pathol Exot. (2013) 106:264–71. doi: 10.1007/s13149-013-0307-8 24072421

[83] WHO. (2020). Primary Health Care Systems (PRIMASYS)-Case Study from Ethiopia, 2017. Available at: https://apps.who.int/iris/handle/10665/341083 (Accessed May 25, 2023).

